# Discovering hidden biodiversity: the use of complementary monitoring of fish diet based on DNA barcoding in freshwater ecosystems

**DOI:** 10.1002/ece3.1825

**Published:** 2015-12-29

**Authors:** Hyunbin Jo, Marc Ventura, Nicolas Vidal, Jeong‐Soo Gim, Teresa Buchaca, Leon A. Barmuta, Erik Jeppesen, Gea‐Jae Joo

**Affiliations:** ^1^ Department of Integrated Biological SciencePusan National UniversityBusan46241South Korea; ^2^ Centre for Advanced Studies of BlanesSpanish National Research Council (CEAB‐CSIC)17300BlanesCataloniaSpain; ^3^ Department of BioscienceAarhus UniversityVejlsøvej 258600SilkeborgDenmark; ^4^ Sino‐Danish Centre for Education and Research (SDC)BeijingChina; ^5^ School of ZoologyUniversity of TasmaniaPrivate Bag 5, Hobart, Tasmania 7000Australia

**Keywords:** DNA barcoding, fish diet, monitoring tool, shallow lakes

## Abstract

Ecological monitoring contributes to the understanding of complex ecosystem functions. The diets of fish reflect the surrounding environment and habitats and may, therefore, act as useful integrating indicators of environmental status. It is, however, often difficult to visually identify items in gut contents to species level due to digestion of soft‐bodied prey beyond visual recognition, but new tools rendering this possible are now becoming available. We used a molecular approach to determine the species identities of consumed diet items of an introduced generalist feeder, brown trout (*Salmo trutta*), in 10 Tasmanian lakes and compared the results with those obtained from visual quantification of stomach contents. We obtained 44 unique taxa (OTUs) belonging to five phyla, including seven classes, using the barcode of life approach from cytochrome oxidase I (COI). Compared with visual quantification, DNA analysis showed greater accuracy, yielding a 1.4‐fold higher number of OTUs. Rarefaction curve analysis showed saturation of visually inspected taxa, while the curves from the DNA barcode did not saturate. The OTUs with the highest proportions of haplotypes were the families of terrestrial insects Formicidae, Chrysomelidae, and Torbidae and the freshwater Chironomidae. Haplotype occurrence per lake was negatively correlated with lake depth and transparency. Nearly all haplotypes were only found in one fish gut from a single lake. Our results indicate that DNA barcoding of fish diets is a useful and complementary method for discovering hidden biodiversity.

## Introduction

Freshwater ecosystems are currently the most threatened systems in the world (Sala et al. 2000). Accordingly, it is important to detect and assess environmental changes in order to ensure proper management and conservation of these valuable ecosystems (Robertson et al. 2012). Classic quantitative techniques (i.e., Surber samplers or dredges) usually applied to monitor the aquatic communities provide useful information for managers. However, these techniques can be limited by biased sampling and incomplete identification (Maroneze et al. 2011). Consequently, barcoding of environmental DNA (eDNA) has been suggested as a new complementary or alternative tool in ecological monitoring (Taberlet et al. 2012; Yoccoz 2012).

Use of eDNA for biological monitoring has increased in recent years. This technique was first described by Ogram et al. (1987) who extracted microbial DNA from the sediment and, today, several papers are available describing the use of eDNA in analyses of soils, waters, and even air (e.g., Taberlet et al. 2012). Andersen et al. (2011) examined the possibility of monitoring large mammals using eDNA soil samples, and eDNA‐based monitoring of fish (Minamoto et al. 2012; Thomsen et al. 2012a,b) and amphibians (Ficetola et al. 2008; Goldberg et al. 2011) has been successful. An alarm system for control of biological invasion of Asian carp has been developed by the U.S. Army Corps of Engineers using water eDNA (Darling and Mahon 2011). Finally, Baird and Hajibabaei (2012) have suggested the use of Biomonitoring 2.0, a descriptive protocol based on eDNA information, in biomonitoring and ecosystem assessments.

While eDNA analyses have been useful in soil and water, analysis of gut and fecal material in living organisms might also be valuable as these organisms by feeding in different areas of the water body integrate spatial (especially mobile forms such as fish) and to some extent also temporal variations. Recently, Schnell et al. (2012) and Calvignac‐Spencer et al. (2012) made an advance in identifying local mammal diversity from mammal blood DNA extracted from terrestrial leeches and carrion flies, respectively. In freshwater habitats, Jo et al. (2014) showed that DNA‐based approaches permit species level identification and revelation of hidden biodiversity as exemplified by analysis of chironomids in the guts of the generalist predator fish *Micropterus salmoides*.

Several other papers have shown that gut contents of fish can be used to supplement biodiversity inventories of benthic macroinvertebrates established using classic visual quantification (Callisto et al. 2002; Tupinambás et al. 2007; Maroneze et al. 2011; Cook and Bundy 2012). One of the few investigations examining the potential of using DNA analyses of fish gut contents in the monitoring of ecosystem function is the study of metabarcoding metazoan diversity of coral reef fish (Leray et al. 2013). Previous studies based on DNA analyses have principally focused on gut content analysis, or on food web composition (Pompanon et al. 2012; De Barba et al. 2014); however, none of these studies have examined the effectiveness of the procedure for monitoring or evaluating biodiversity.

We applied sequence‐based DNA barcoding to determine the diet of a generalist predator (brown trout, *Salmo trutta*) from gut contents and compared the results with visual quantification data. On the basis of our results, we discuss the potential of using prey organisms in fish gut contents as a supplementary monitoring tool to reveal hidden biodiversity.

## Materials and Methods

### Study sites

Tasmania was selected to test the ability of using fish gut contents as a monitoring tool because it is geographically and genetically isolated and has a unique flora and fauna. Since 1864, nine, mainly European, fish species have been introduced to the island's freshwaters for angling purposes (Lintermans 2004). The introduced species coexist with the native fish fauna, which is composed mainly of galaxiids (16 species, most of which are considered threatened, Hardie et al. 2006). The most successful introduced species is brown trout, a generalist predator whose diet reflects the prevailing habitat conditions (Kawaguchi and Nakano 2001).

### Fish sample collection and storage conditions

Brown trout were sampled in 10 lakes in Tasmania during the austral summer (February) in 2007 using Nordic gill nets with 14 different mesh sizes ranging from 6.25 to 75 mm from knot to knot and fyke nets. The nets were set overnight (17–20 h in total) in the littoral and pelagic zones, the number of gill nets used depended on lake size and ranged from 2 to 4 nets per lake, while fyke nets were placed near the shore in all cases. Fish density was calculated as CPUE (Catch per Unit Effort, number of fish per gill net^−1^ h^−1^ or number of fish per fyke net^−1^ h^−1^). Brown trout were measured to total length (0.1 cm) and weighed (g). After capture, the guts were removed and preserved in 96% ethanol for visual quantification and laboratory DNA analysis.

The ethanol‐preserved gut samples were transferred from Tasmania, Australia, to Denmark and stored at room temperature. In December 2012, the samples were sent to China for visual quantification and finally to South Korea where all samples were freeze stored (−80°C) for DNA analysis. The samples were removed from the ethanol solution three times during transfer and analysis (Table S1). This procedure is not optimal for DNA analyses but provides information on the usability of stored and preanalyzed samples for obtaining postsampling eDNA data to substitute or complement traditional taxonomic information based on visual gut content analysis.

### Physicochemical parameters and visual quantification

Depth‐integrated water samples were taken at the deepest point in the lakes and later analyzed in the laboratory for nutrient concentrations and phytoplankton chlorophyll *a* (Chl‐*a*). In addition, depth profiles of water temperature, dissolved oxygen, pH, and conductivity were recorded and Secchi depth was measured in situ (N. Vidal Pers. Comm.). The Chl‐*a* concentration was used as a measure of phytoplankton biomass, 100–1000 mL water samples were filtered through Whatman GF/C filters (47 mm in diameter) depending on concentration. Chlorophyll *a* was determined spectrophotometrically after ethanol extraction (Jespersen and Christoffersen 1987). Total phosphorus (TP) was determined as molybdate reactive phosphorus (Murphy and Riley 1962) following persulfate digestion (Koroleff 1970) and total nitrogen (TN) as nitrite after potassium persulfate digestion (Solorzano and Sharp 1980).

Gut samples from 148 brown trout were examined under a stereomicroscope (Zeiss, Stemi 1000) at 20–100× magnification in 2012. The prey were identified to species level or the lowest taxonomic level possible and counted, and each item was determined volumetrically using the water replacement method for big items and a graduated slide for small ones. We analyzed 102 of 148 gut samples for identification of ingested diet items using DNA analysis. The remaining samples (46 guts) were empty. Detailed information of gut samples is given in Table S2.

### DNA extraction and amplification

The gut contents were removed from the fish guts and stored in 96% ethanol in the field followed by visual quantification in the laboratry. After visual quantification of the gut contents, ethanol was completely volatilized from the samples preceding the DNA extraction process. The gut contents were then frozen in liquid nitrogen and homogenized manually using a mortar and pestle. Each gut sample was totally homogenized and 25 mg was removed for genomic DNA analysis, and the remaining homogenized samples were stored in a freezer at −80°C. The 25 mg subsample was isolated using LaboPass Tissue Miniprep kit (*n* = 102; Cosmogenetech, Seoul, Korea), Qiagen Stool DNA kit (*n* = 6), and Qiagen DNA extraction kit (*n* = 28; Qiagen, Hilden, Germany) according to the manufacturer's directions. Between homogenization of each gut content sample, the mortar and pestle were cleaned using de‐ionized water and any remaining material was burnt off using methanol to prevent cross contamination of samples.

PCR amplification was performed using AccuPower Hot start PCR PreMix (Bioneer) with genomic DNA and primers in a final volume of 20 *μ*L. The COI region was amplified with LCO1490 (5′‐GGTCAACAAATCATAAAGATATTGG‐3′) and HCO2198 (5′‐TAAACTTCAGGGTGACCAAAAAATCA‐3′) (Folmer et al. 1994). The PCR thermal regime consisted of one cycle of 10 min at 94°C; 40 cycles of 1 min at 94°C; 1.5 min at 50°C; 1 min at 72°C; and a final cycle of 5 min at 72°C in a Mastercycler (Eppendorf, Hamburg, Germany). PCR products were separated using 1.5% agarose gels. After purification using Labopass Gel Extraction kit (Cosmogenetech), cloning was carried out using the pGEM‐T easy vector (Promega, Madison, WI).

Cloned plasmid DNA was isolated according to the alkaline lysis method using Labopass Plasmid Miniprep kit (Cosmogenetech). Individually isolated plasmid DNA was then digested using the restriction enzyme *Eco*RI to confirm insertion. Positive clones for each sample were analyzed to species‐specific sequences with SP6 primers using an automated 3730 DNA analyzer (Applied Biosystems, Foster City, CA). Whenever there were more than 10 clones available from each gut sample, we used a rarefaction curve (PAST program; Hammer et al. 2001) to determine the exact number required. On the basis of the results from the rarefaction curve analysis, it was determined that the minimum number of clones required was 10 for each sample. When the rarefaction curve did not reach a constant value (i.e., identification of more than three operational taxonomic units, OTUs) within the first 10 clones, we repeated the process with another 10 clones, and this process was repeated until all OTUs from the samples were identified.

### DNA sequence analysis and statistics

Sequence alignment was performed using Clustal W 2.0 (Larkin et al. 2007). A BLASTn (Altschul et al. 1990) search was performed to identify sequences with the best hits. Ten sequences of the top hits from the NCBI (Benson et al. 2005) and BOLD systems (Ratnasingham and Hebert 2007) database, in addition to two or three outgroups from the nearest families, were downloaded. The degree of similarity between the obtained sequences was assessed using the maximum likelihood (ML) algorithm (Saitou and Nei 1987) as implemented in MEGA 6.0 (Tamura et al. 2013). The degree of information redundancy in fragments compared using ML was assessed by bootstrap resampling of 1000 pseudoreplicate datasets (Felsenstein 1985).

To relate each OTU to a previously sequenced species, we adopted two criteria that according to Jo et al. (2014) ensure accurate species identification. The first criterion was acceptance of a species name if the given OTU had ≥98% compliance with a known species. This first criterion assumes that a 2% difference between an OTU and a known species may be caused by intraspecific variation or PCR and sequencing errors (Jarman et al. 2004; Clare et al. 2009). The second criterion was that in the constructed phylogenetic tree the putative known species and the given OTU should appear within the same cluster (Fig. S1).

The number of haplotypes and the nucleotide diversity per fish gut were calculated using DNASP 5.0 (Rozas et al. 2003). The levels of genetic diversity among lakes, among fish guts within each lake, and between the individual fish guts were analyzed with a hierarchical analysis of molecular variance (AMOVA; Excoffier et al. 1992) with ARLEQUIN3.5 (Excoffier and Lischer 2010). We used Kimura two parameter distance to calculate the distance among haplotypes to infer genetic differentiation (*F*_ST_).

To reveal if genetic diversity was related to the main environmental gradients we first characterized the lake environmental characteristics with a principal component analysis (PCA). Next, we analyzed for nonlinear relationships between the environmental variables (lake area, altitude, depth, Secchi depth, temperature, dissolved oxygen, conductivity, pH, total phosphorus, total nitrogen, and Chl‐*a*) and the number of OTUs, the number of haplotypes, and nucleotide diversity found in the gut contents of each lake using linear regression, and compared rarefaction curves with lakes ordered according to the different environmental parameters to reveal if species occurrences were constrained by environmental gradients. Statistical significance was evaluated at *α* = 0.05. We used PASW Statistics 18 (SPSS Inc., Chicago, IL) for the statistical analysis.

## Results

### Gut content analyses based on DNA barcoding

DNA barcoding analysis returned a list of consumed diet items with overall good resolution. From the 148 gut samples, 46 samples were completely empty. DNA material from 47 of the remaining 102 gut samples was successfully amplified by PCR (48.0% success rate; Table S2). We obtained 414 robust 658‐bp sequences (Table S3); the clones contained DNA from 44 unique sequences from different species following the criteria described in the Materials and Methods section (see above). Among these, only 14 OTUs were clearly identified to species level when compared with the NCBI and Bold systems database (31.8% of 44 diet OTUs), indicating that most of the sequences belonged to species that have not been previously sequenced.

**Figure 1:**
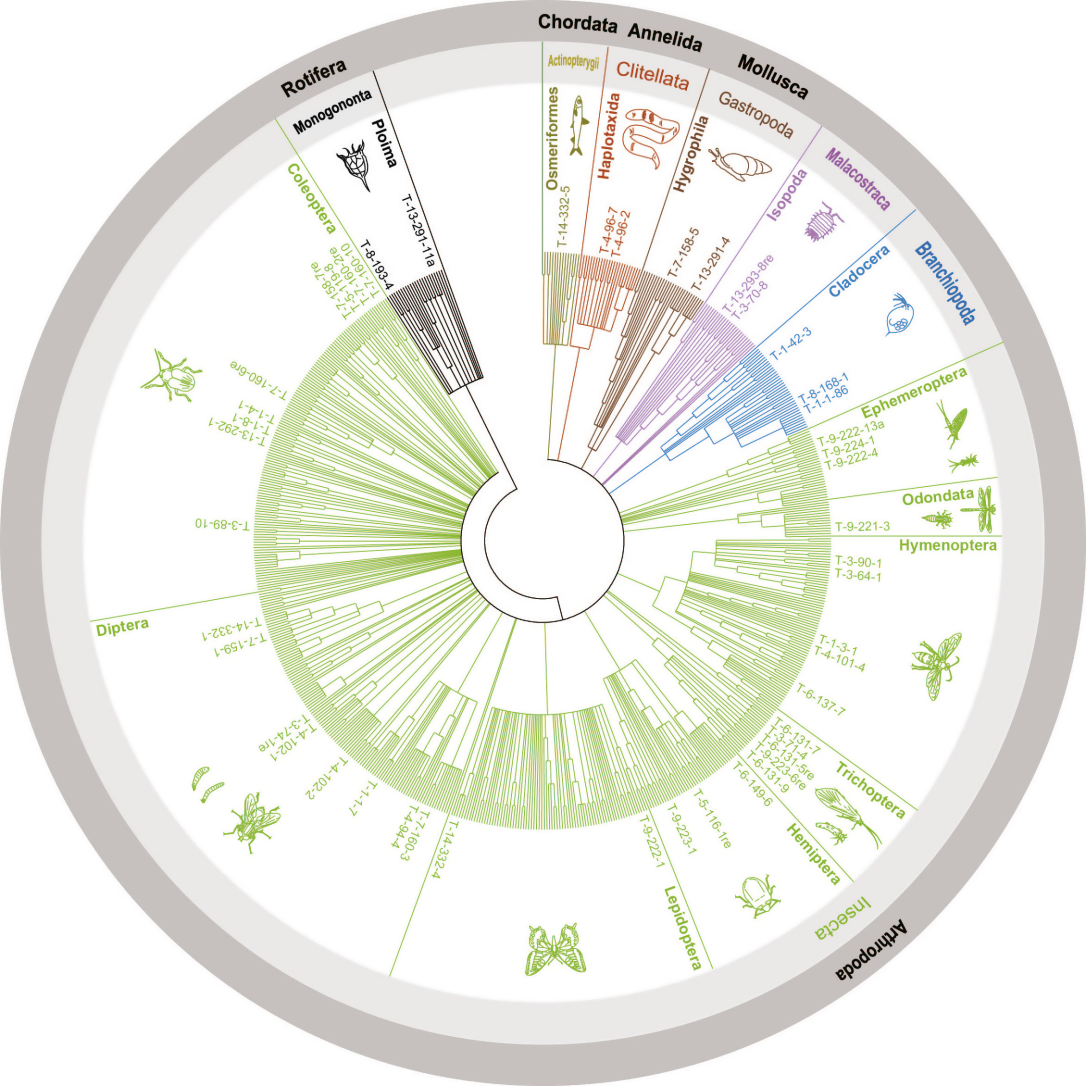
Circular phylogenetic tree showing a broad range of diet OTUs from gut contents.

Following adoption of the identification criteria (see Materials and Methods), we determined OTUs belonging to five phyla, including seven classes, 14 orders, and 24 families, based on NCBI and Bold systems database searches and phylogenetic tree construction (Figs 1 and S1 and Table S3 with raw data). Insecta comprised the largest proportion of the identified OTUs (32 OTUs, 72.7% of total OTU number) followed by Branchiopoda, Malacostraca, Gastropoda, Clitellata, and Monogononta in the gut contents. In comparison, we only found one fish OTU, *Galaxias maculatus*, an endemic species in Tasmania (2.3% of the OTUs).

The alignment of 414 Tasmanian sequences of mtDNA contained 66 variable nucleotides of a total of 658. In total, we detected 246 haplotypes in the Tasmanian lakes. Nearly all haplotypes were found in only one fish gut from one lake (93% of the haplotypes), 5.3% were found in two fish from two different lakes, 1.2% in three fish from three different lakes, while only 0.4% of the haplotypes were found in two different fish from the same lake. The number of haplotypes in each gut sample was highly correlated with the number of sequenced clones (*R* ^2^ = 0.906, *P < *0.001). The OTUs with the highest proportion of haplotypes were the families of terrestrial insects Formicidae, Chrysomelidae, and Torbidae and the freshwater Chironomidae. Among all haplotypes, 234 were singletons, while the remaining 12 haplotypes occurred just two or three times (Table 1). AMOVA revealed a relatively low genetic variability between the lakes (9.6%). Most of the genetic variation was between species (65.5% of the total genetic variation) and 24.9% within species, and all values were highly significant (*P *<* *0.001; Table 2). The average number of nucleotide differences between sequences was *k *=* *165.68, yielding an overall nucleotide diversity of *Pi* = 0.319.

**Table 1:** List of diet items (OTUs) sequenced from trout gut contents using DNA barcoding (COI: 658bp)

Order	Family	Genus + Species	No. clones	No. haplotypes	Identity	Query	Access ID	Level
Osmeriformes	Galaxiidae	*Galaxias maculatus*	11	6	99	100	AP004104.1	Species
Isopoda	Phreatoicidae	*Colubotelson* sp.	4	3	89	88	AF255775.1	Genus
	Asellidae	*Asellus* sp.	15	9	89	98	AY531829.1	Genus
Cladocera	Daphniidae	*Daphnia longicephala*	2	2	98	98	AF217114.1	Species
		*Daphnia laevis*	1	1	99	100	KC616964.1	Species
		*Daphnia* sp. 1.	1	2	92	100	KC616937.1	Genus
Ephemeroptera	Heptageniidae	Heptageniidae 1.	13	7	78	91	KM444914.1	Family
		Heptageniidae 2.	12	8	83	100	GU713795.1	Family
Odonata	Lestidae	*Austrolestes* sp.	6	3	90	98	KF369320.1	Genus
Hemiptera	Belostomatidae	*Appasus japonicus*	1	1	99	100	AB742657.1	Species
	Notonectidae	Notonectidae	15	5	86	99	KM022030.1	Family
	Corixidae	Corixidae	10	3	90	100	KM021675.1	Family
Hymenoptera	Apidae	*Apis mellifera*	2	2	98	100	KM458618.1	Species
	Formicidae	*Camponotus hartogi*	20	10	97	100	JN134876.1	Species
		*Camponotus* sp.	8	2	91	100	JN134876.1	Genus
		*Iridomyrmex* sp.	16	10	91	100	JN134882.1	Genus
		Formicidae	19	15	86	99	JQ083703.1	Family
Coleoptera	Dytiscidae	Dytiscidae	8	4	87	100	KF714816.1	Family
	Chrysomelidae	Chrysomelidae 1.	2	3	85	99	KJ962415.1	Family
		Chrysomelidae 2.	15	7	86	99	KM439764.1	Family
		Chrysomelidae 3.	32	15	85	99	KM439873.1	Family
	Coccinellidae	Coccinellidae	1	1	87	100	KM441829.1	Family
	Staphylinidae	Staphylinidae 1.	6	4	83	100	KM444549.1	Family
		Staphylinidae 2.	12	11	83	100	KM441364.1	Family
		Coleoptera	44	22	85	99	KM448041.1	Order
Diptera	Chironomidae	*Chironomus oppositus*	1	1	99	100	KJ946672.1	Species
		*Riethia stictoptera*	13	10	98	100	KC750518.1	Species
		*Procladius villosimanus*	16	14	99	95	HQ248026.1	Species
		*Cryptochironomus* sp.	1	1	93	100	KJ946724.1	Genus
		*Chironomus* sp.	1	1	92	100	KC750309.1	Genus
		*Anatopynia* sp.	36	20	91	95	HQ247986.1	Genus
		*Coelopynia* sp.	8	6	93	100	KC750362.1	Genus
		Diptera	4	1	85	99	KF401606.1	Order
Trichoptera	Leptoceridae	*Oecetis australis*	1	1	99	100	FN601025.1	Species
		*Notalina fulva*	2	1	99	100	FN600985.1	Species
	Atriplectididae	*Atriplectides dubius*	27	17	100	100	FN601034.1	Species
	Philorheithridae	*Aphilorheithrus* sp.	1	1	90	100	FN600945.1	Genus
Lepidoptera	Crambodae	*Hygraula nitens*	2	1	99	100	HQ951670.1	Species
Hygrophila	Physidae	*Physella anatina*	6	4	99	99	AY651177.1	Species
	Planorbidae	*Glyptophysa* sp.	6	4	91	94	EF012179.1	Genus
Haplotaxida	Megascolecidae	Megascolecidae 1.	6	3	85	98	GU014157.1	Family
		Megascolecidae 2.	4	2	82	98	GU014155.1	Family
Plioma		Plioma 1	1	1	78	83	JF714414.1	Order
		Plioma 2	2	1	80	83	DQ089728.1	Order
	Total number of clone sequences		414	246	90.4	98.0		
	Total number of diet items (OTUs)		44					

**Table 2:** Results from the analysis of molecular variance (AMOVA) of the OTUs obtained from fish guts subdivided into three different levels (i) among lakes, (ii) among fish individuals within each lake, and (iii) within fish individuals

Source of variation	df	Sum of squares	Variance components	Percentage of variation	*P*
Among lakes	9	11419	10.4	9.6	<0.001
Among fish within lakes	37	22433	71.3	65.5	<0.001
Within fish individuals	368	10018	27.1	24.9	<0.001
Total	414	43870	108.8		

### Comparison of visual quantification and DNA barcoding

**Figure 2:**
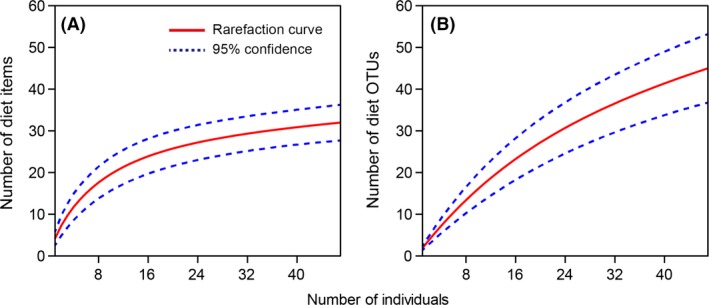
Rarefaction curves (A: visual quantification data, B: DNA barcoding data).

While visual quantification enabled identification of diverse taxa from the gut samples, DNA barcoding had a much higher level of resolution. There was a 1.4‐fold increase in the occurrence of dietary items identified via DNA barcoding than by visual identification (Tables 1 and 3). In most cases visual quantification did not enable total species identification (32 diet items); thus, DNA barcoding gave more taxa and higher taxonomic resolution (44 OTUs) than visual quantification. The comparison of the rarefaction curves indicates saturation of the number of taxa obtained by visual quantification, while DNA barcoding did not indicate any saturation (Fig. 2). DNA barcoding yielded 12‐ and 4.7‐fold increases at the genus and species levels, respectively, enhancing the level of resolution in identification (Table 4).

**Table 3:** List of diet items determined using visual quantification

Phylum	Class (subclass)	Order	Family	Identification name	Level
Chordata	Actinopterygii			Fish (juvenile)	Class
	Amphibia	Anura		Frog	Order
Arthropoda	Malacostraca	Isopoda	Asellidae	Amphipods	Family
			Phreatoicidae	Phreatoicidae	Family
	Branchiopoda	Cladocera	Daphniidae	*Daphnia* sp.	Genus
	Insecta (Pterygota)	Ephemeroptera	Leptophlebidae	Leptophlebidae	Family
			Oniscigastridae	Oniscigastridae	Family
		Odonata		Anisoptera	Order
				Zygoptera	Order
		Hemiptera	Cicadidae	Cicadidae	Family
			Corixidae	Corixidae	Family
			Notonectidae	Notonectidae	Family
				Hemiptera	Order
		Hymenoptera	Apidae	Bee	Family
				Wasp	Family
			Formicidae	Ant	Family
		Coleoptera	Chrysomelidae	*Paropsisterna* sp.	Genus
			Dytisidae	Aquatic Coleoptera	Family
			Scarabaeidae	Anoplognatus	Family
				Coleoptera	Order
		Diptera	Chironomidae	Chironomidae (adult)	Family
				Chironomidae (larvae)	
		Trichoptera	Atriplectididae	Atriplectididae	Family
				Trichoptera (Adult)	Order
				Trichoptera (Pupa)	
		Lepidoptera	Glyphipterigidae	Glyphipterigidae	Family
Mollusca	Gastropoda	Basommatophora	Bullinidae	*Isidorella hainesii*	Species
			Lymnaeidae	*Austropeplea tormentosa*	Species
				Snail	Class
		Sorbeoconcha	Hydrobiidae	*Hydrobia buccinoides*	Species
	Bivalvia	Veneroida	Sphaeriidae	Pisidium	Family
Annelida	Clitellata			Hirudinea	Class
				Anelidea	Phylum
Nematomorpha				Nematomorpha	Phylum
				Partially identified particles	Family
				Unidentified particles	
		Number of diet items	32		

**Table 4:** Comparison of identification level resolution between visual quantification and DNA barcoding

	Visual	%	DNA	%
Phylum	2	6.3	0	0.0
Class	3	9.4	0	0.0
Order	6	18.8	4	9.1
Family	17	53.1	14	31.8
Genus	1	3.1	12	27.3
Species	3	9.4	14	31.8
	32	100.0	44	100.0

At both individual fish and lake levels, the number of diet items obtained from visual quantification was lower than the number of OTUs obtained from DNA barcoding (Figs S2 and S3). When sorted into terrestrial or aquatic origin of taxa, aquatic organisms comprised 27 OTUs (61.4%) and terrestrial organisms 17 OTUs (38.6%). Rather similar results were obtained from visual quantification (using frequency and volume): 37% of the diet consisted of terrestrial organisms and 63% of aquatic organisms. When divided into orders, the richness of Coleoptera and Diptera revealed via DNA barcoding was substantially higher, 2.7‐fold and 7‐fold, respectively, than when using visual quantification. Lepidoptera, Hygrophila, Haplotaxida, and Plioma were found only via DNA barcoding. However, Sorbeoconcha and Veneroida were not detected by DNA barcoding.

### Relationship with environmental parameters

Most of the study lakes were shallow with an average maximum depth (m) of 3.1 ± 1.2 (mean and standard deviation), and area and altitude covered a wide range from 24 to 4433 ha and 467 to 1164 m, respectively. Trophic state ranged from oligotrophic (0.002 mg L^−1^ TP) to eutrophic (0.122 mg L^−1^ TP); accordingly, Chl‐a ranged between 0.3 and 36.3 *μ*g L^−1^. Conductivity was below 100 *μ*S cm^−1^ in all lakes (Table 5). Most lakes were oligotrophic, which correlated with lake area (larger and more eutrophic lakes were found at lower altitudes) (PCA; Fig. S4).

**Table 5:** Physicochemical characteristics of the study sites (temperature: Temp, dissolved oxygen concentration: DO, conductivity: Cond, total phosphorous concentration: TP, total nitrogen concentration: TN, phytoplankton chlorophyll a concentration: Chl‐a, capture per unit effort: CPUE)

Lake name	Area (ha)	Altitude (m)	Depth (m)	Secchi (m)	Temp (°C)	DO (mg L^−1^)	Cond (*μ*S cm^−1^)	pH	TP (mg L^−1^)	TN (mg L^−1^)	Chl‐*a* (*μ*g L^−1^)	CPUE
Lake Sorell	4433	818	2.7	0.2	17.7	7.6	99	7.4	0.12	1.25	10.2	0.63
Lake Ada	223	1158	1	1	16.8	7.7	27	7.8	0.01	0.40	3.3	0.90
Lake Augusta	450	1150	0.8	0.8	19.5	7.8	21	7.4	0.01	0.34	1.3	0.85
Howes Bay Lagoon	24	1164	0.5	0.5	17.2	9.6	36	6.9	0.02	0.55	3.2	0.50
Lake Bronte	468	676	5.5	3.1	21.0	7.4	29	6.9	0.01	0.24	0.3	0.56
Carter Lake	24.3	1163	1	1	19.9	7.9	34	7.3	0.01	0.51	2.1	0.50
Lake Echo	4030	867	13.1	4.5	17.9	7.9	28	7.1	0.01	0.25	1.8	1.41
Penstock Lagoon	160	938	1.5	1.5	20.0	7.3	57	7.3	–	–	1.8	0.43
Lake Leake	676	572	2.5	2.1	19.4	7.6	68.7	7.0	–	–	4.2	0.55
Lake Tooms	656	467	2.1	0.6	17.1	7.6	98.9	8.2	0.09	1.30	36.3	0.50
Average	1114.4 ± 525.5	897.3 ± 83.2	3.1 ± 1.2	1.5 ± 0.4	18.7 ± 0.5	7.8 ± 0.2	49.9 ± 9.4	7.3 ± 0.1	0.03 ± 0.02	0.61 ± 0.15	6.4 ± 3.4	0.68 ± 0.30

**Figure 3:**
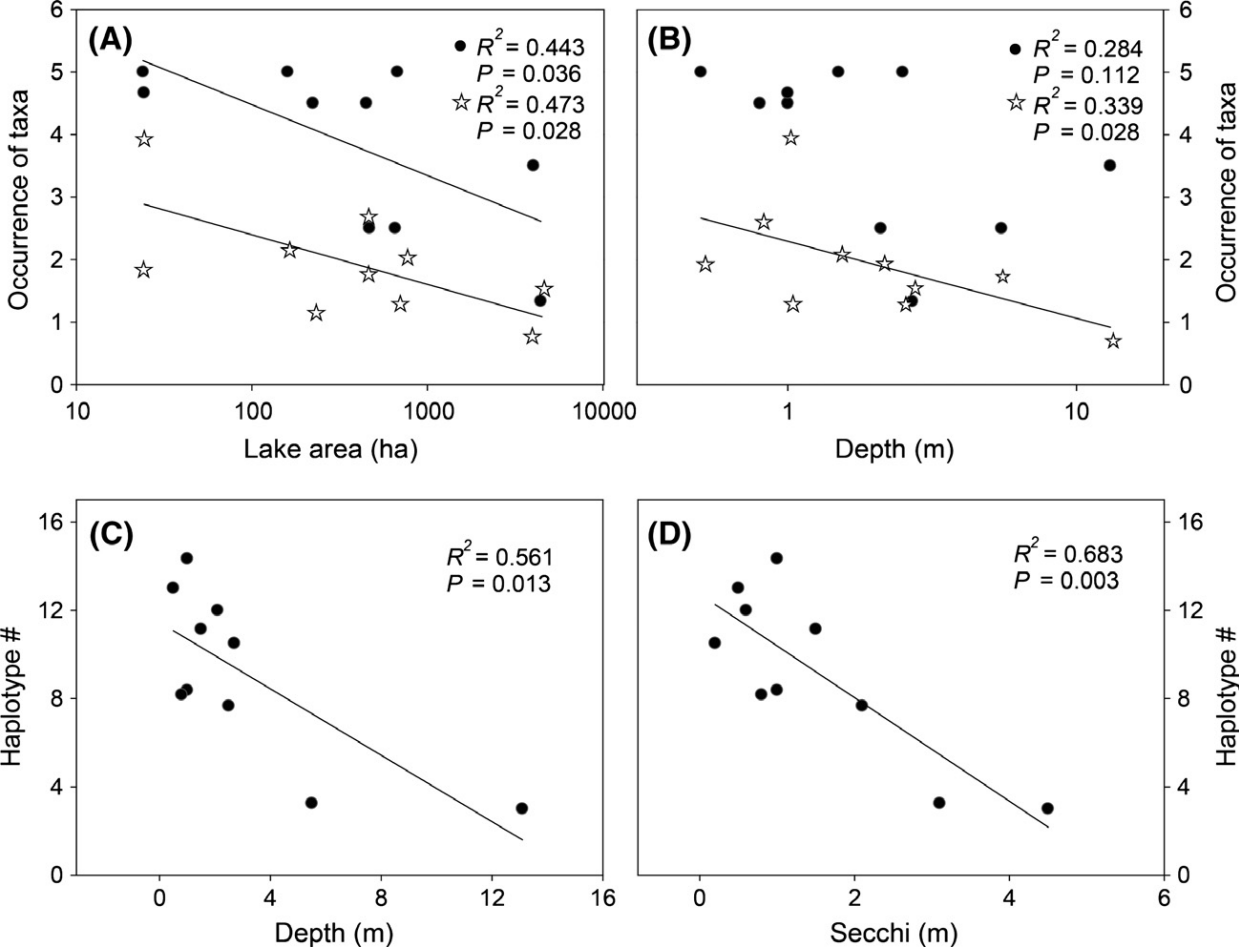
Relationships between the average number of taxa identified with visual quantification (black circles) and DNA barcoding (OTUs; stars) with lake surface area (A) and lake depth (B). Relationship between the average number of haplotypes per lake and lake depth (C) and Secchi depth (D).

Regression analysis identified a nonlinear relation between the number of diet items in the fish guts and lake area or depth (logarithm transformed) using both methodological approaches. The occurrence of visually inspected taxa found in each lake decreased gradually (*R* ^2^ = 0.443, *P *=* *0.036) with increasing lake area (Fig. 3A). The number of diet OTUs (*R* ^2^ = 0.473, *P *=* *0.028) decreased with both increasing lake area and depth (*R* ^2^ = 0.339, *P *=* *0.028; Fig. 3A,B).

The average number of haplotypes per lake showed a negative relationship with lake depth (*R* ^2^ = 0.561, *P *=* *0.013*;* Fig. 3C) and Secchi depth (*R* ^2^ = 0.683, *P *=* *0.003; Fig. 3D). Nucleotide diversity in the lakes was related negatively to TP (*R* ^2^ = 0.227, *P = 0.233*) and temperature (*R* ^*2*^ = 0.201, *P *=* *0.194). The proportion of terrestrial organisms occurring in each gut sample when using visual quantification showed no relationships with physicochemical factors except for a positive relationship with lake area (*R* ^2^ = 0.469, *P *=* *0.029).

**Figure 4:**
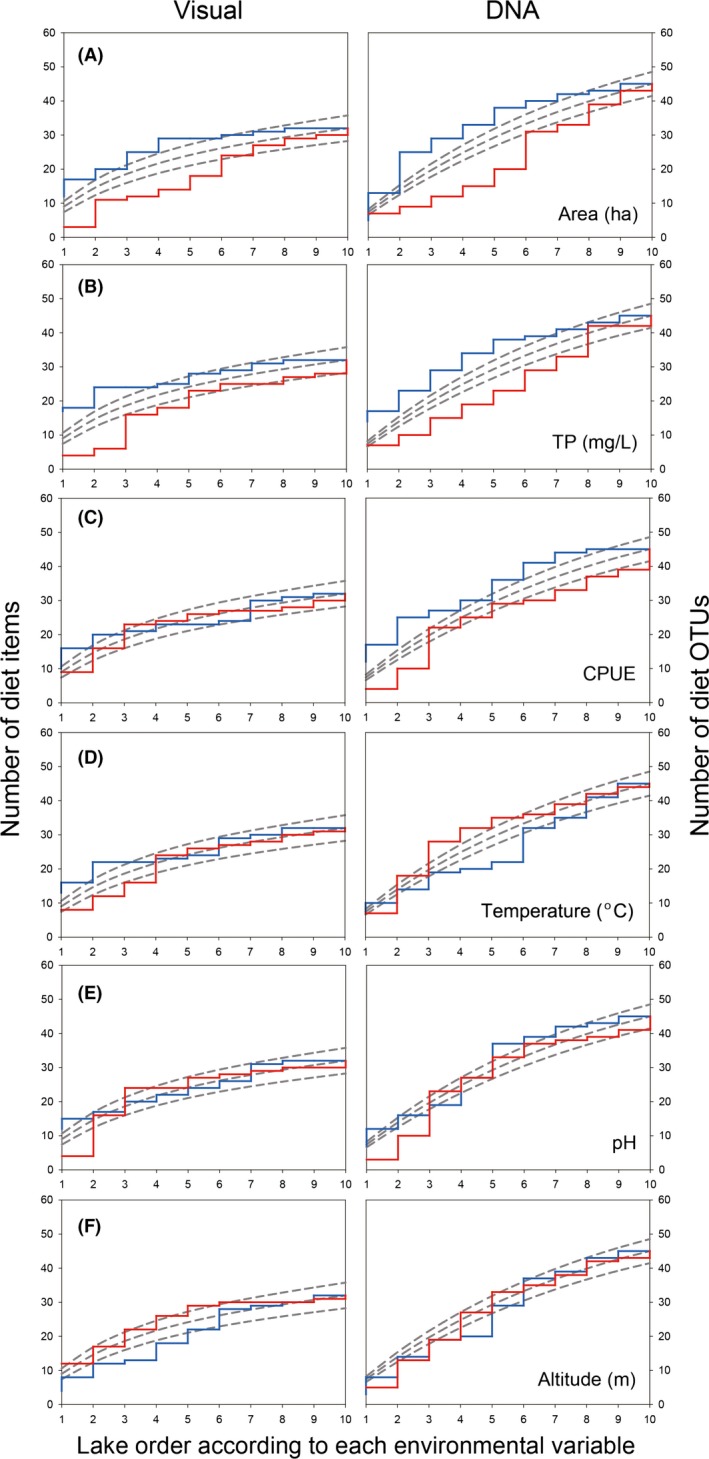
Accumulation curve following the order of ecological factors with rarefaction curve (blue: increasing prey species, red: decreasing prey species) for visually identified taxa (left panels) and DNA barcoding OTUs (right panels) (A: Area, B: TP, C: CPUE, D: Temperature, E: pH, F: Altitude).

Different patterns emerged in the rarefaction curve analysis between visual quantification and DNA barcoding. Figure 4 shows the environmental variables and their associated accumulation curves arranged from the largest to smallest accumulation values of DNA barcoding. Differences in accumulation values occurred based on the methodological practice applied. In general, the number of taxa identified using DNA barcodes was greater than the taxa identified using visual quantification.

## Discussion

### The potential of using DNA barcoding of fish gut contents as a monitoring parameter

Our results showed that DNA barcoding of the gut contents of a generalist fish predator holds potential as a monitoring tool. In our study of shallow lakes from Tasmania, DNA barcoding yielded more detailed information on the food choices of fish than did traditional gut content analyses and revealed hidden biodiversity, as well. The DNA barcoding results demonstrated higher identification resolution than visual quantification (Table 4, Figs S2 and S3). Our analysis identified 53.8% of known aquatic orders despite the small number of samples (*n* = 47). The number of taxa identified by DNA barcoding was 1.4‐fold higher than by visual identification. When grouped by order, the diversities of coleopterans and dipterans identified via DNA barcoding were 2.7‐fold and 7‐fold higher, respectively, compared to visual quantification. Moreover, our results included not only aquatic organisms but also multiple terrestrial organisms.

Maroneze et al. (2011) have earlier demonstrated the potential of using fish as an ecological indicator as a supplement to biodiversity inventories of benthic macroinvertebrates based on classic visual quantification as the identification resolution (i.e., family or class) of visual quantification may affect the assessment results. Hawkins et al. (2000), for example, demonstrated that a complete taxa list, based on genus/species identification, detected the effects of watershed alteration on stream invertebrate assemblages in the Sierra Nevada of California, whereas family‐based identification did not reveal any differences among sites. Therefore, improved identification resolution (i.e., species or genus level) based on DNA barcoding, as in our study, may be effective in ecosystem assessments.

Kawaguchi and Nakano (2001) found that determination of terrestrial invertebrates in fish diets provided information about the local distribution of salmonids in a headwater stream. We found a gradual decrease in the number of taxa in fish stomachs with increasing lake area and depth using both the visual quantification and the DNA barcoding approach (Fig. 3). Our study showed that the diet of brown trout consists of both aquatic and terrestrial organisms, aquatic organisms comprising 27 OTUs (61.4%) and terrestrial organisms 17 OTUs (38.6%). Rather similar results were obtained by visual quantification (using frequency and volume) where 37% of the diet was found to consist of terrestrial organisms and 63% of aquatic organisms.

We found a relationship between number of diet items and environmental factors. Specifically, lake area and depth had a negative relationship with the number of diet items which may be attributed to decreasing heterogeneity (less importance of the shallow and often macrophyte covered littoral zone) (Jeppesen et al. 1998). In addition, the larger lakes were also the most eutrophic ones which also might reduce animal diversity (Vadeboncoeur et al. 2001; Liboriussen and Jeppesen 2003). Our results further showed that the DNA barcoding approach showed higher molecular variability (in number of haplotypes or nucleotide diversity), each fish having OTUs with different haplotypes. Specifically, in all lakes, the average number of haplotypes showed a negative relationship with lake depth and Secchi depth, while nucleotide diversity correlated with TP and temperature, emphasizing the capacity of the method to track not only taxonomical but also genetic changes related to changes in the environment. Our results thus indicate that analyses of fish gut content, combining visual quantification with DNA barcoding, could be an effective and complementary monitoring and assessment tool for lakes.

### Effectiveness of the DNA barcoding approach

We focused on the use of gut content analysis as a method for the ecological monitoring of aquatic biodiversity. We attempted to assess the effectiveness of the procedure as a monitoring tool for biodiversity in lakes. Our results indicate that DNA barcoding of fish gut contents provides robust species level identification (Table 1, Fig. S1). The classic universal primers set (LCO1490, HCO2198) proved to be efficient for identification, as has also been demonstrated in other studies (e.g., Hebert et al. 2003; Ward et al. 2009; Ekrem et al. 2010). Moreover, DNA barcoding is based on a wide‐ranged database (i.e., Barcoding of Life Initiative, Fig. 1) on aquatic and terrestrial organisms, allowing comparison of DNA sequences for most categories of animal taxa (Ratnasingham and Hebert 2007), and DNA barcoding proved to be more efficient at revealing hidden biodiversity than classic visual quantification. Another benefit is that fish gut contents provide more fresh DNA material than other environmental (i.e., soil, water) and fecal material and that the samples are easy to preserve, requiring only 95–99% ethanol and storage at room temperature. Moreover, the different fish species may exploit different environments and potentially together represent a broad range of prey if fish species with different trophic niches are included in the analysis. The relatively low species level identification resolution observed in our study was likely due to incompleteness of the Tasmanian NCBI and Bold systems database. This is of concern because we cannot be certain if prey species are rare or endemic if they have not been sequenced. Tasmania is known for its high level of endemism for terrestrial and aquatic species (Cracraft 1991; Hardie et al. 2006), which constitutes a problem in the use of DNA barcoding as Tasmanian lakes are poorly represented in the NCBI and BOLD systems database. Therefore, we encourage the establishment of a complete DNA barcoding database for Tasmanian freshwaters, which may potentially reveal new species.

### Pitfalls and further study

There was relatively low coherence between the visually based and DNA‐based results, both within lakes and in the individual gut samples (Figs S2 and S3). This may, in part, be attributed to the handling of samples and the prolonged storage of the samples upon field collection until analyses. In addition, we used a long barcoding region and one set of classical Folmer primers (658 bp; LCO1490, HCO2198) as identifier regions for the test. The length of the segment can easily lead to degradation or fragmentation, and some taxonomic groups are not adequately covered in the database when it comes to the classic DNA region. Our results showed that some taxonomic groups such as Sorbeoconcha and Veneroida were not detected by DNA barcoding. Deagle et al. (2014) suggested a multiplexing metabarcoding approach applying group‐specific markers (multiple primers sets) to overcome this problem. Since brown trout are known to be generalist carnivores (Kawaguchi and Nakano 2001), their stomach contents are more easily analyzed using one set of universal primers. However, omnivores or herbivorous organisms require multiple primers sets in order to determine any unknown material (De Barba et al. 2014). Despite these difficulties, we obtained a higher track number of OTUs than when using only classic visual quantification techniques. Particularly soft‐bodied animals lacking chitin for preservation during digestion may be better tracked by eDNA methods. A final limitation of using DNA barcoding in fish diet analyses is the lack of information on quantitative gut and fecal material. While this limitation may be problematic when using DNA cloning, the use of more technologically advanced procedures such as quantitative PCR and next‐generation sequencing (NGS) diminishes this problem, yielding greater quantification certainty. The studies available using these more advanced methods such as NGS of stomach content of fecal material in food‐web studies provided more powerful biodiversity estimates (Pompanon et al. 2012; De Barba et al. 2014). These methods have the disadvantage of more sophisticated bioinformatic methods. Our approach has shown that if the NGS approach is not feasible, using DNA cloning provides clear superior results than those reported by visual quantification alone. In contrast, classic visual quantification allows for discrimination between adults and juveniles/larvae and provides quantitative information for application in gut and fecal analysis. When using the current method, we therefore recommend a combination of visual quantification and DNA barcoding for gut content analysis; however, with the more detailed analytical procedures available today, the need for visual identification is no longer pressing.

## Data Accessibility

DNA sequences: DRYAD entry doi:10.5061/dryad.tj0hj.

## Conflict of Interest

None declared.

## Supporting information


**Figure S1** Alignment of the sequences obtained in this study with all the sequences retrieved from the Genbank and BOLD system for the COI region.

**Figure S2** Comparison of the number of identification levels between visual quantification and DNA barcoding for all the sampled lakes, first column: visual quantification; second column: DNA barcoding.
**Figure S3** Comparison of the number of identification levels between the visual quantification and DNA barcoding analysis for each individual fish (individual length information given in Table S1 in accordance with *x*‐axis legend numbers); first column: visual quantification, second column: DNA barcoding.
**Figure S4** Visual presentation of the first two PCA axes summarizing environmental variables and the lakes (lake information provided in Table 2).
**Table S1** History of sample information (room temperature: RT).
**Table S2** Study sites, sample, and PCR amplification information.
**Table S3** Raw data on *Salmo trutta* diet based on DNA barcoding analysis.
**Table S4** Average nucleotide diversity (ND).
