# Supplementary material for: Discovering hidden biodiversity: the use of complementary monitoring of fish diet based on DNA barcoding in freshwater ecosystems
Source: Ecol Evol. 2015 Dec 29;6(1):219–32. doi: 10.1002/ece3.1825 (PMC4716507; doi:10.1002/ece3.1825)
Supplement: Supplementary file 2 — Figure S2 Comparison of the number of identification levels between visual quantification and DNA barcoding for all the sampled lakes, first column: visual quantification; second column: DNA barcoding. Figure S3 Comparison of the number of identification levels between the visual quantification and DNA barcoding analysis for each individual fish (individual length information given in Table S1 in accordance with x‐axis legend numbers); first column: visual quantification, second column: DNA barcoding. Figure S4 Visual presentation of the first two PCA axes summarizing environmental variables and the lakes (lake information provided in Table 2). Table S1 History of sample information (room temperature: RT). Table S2 Study sites, sample, and PCR amplification information. Table S3 Raw data on Salmo trutta diet based on DNA barcoding analysis. Table S4 Average nucleotide diversity (ND). [file ECE3-6-219-s002.doc]

*Fig. S1. 🡪 20150216_Figure S1.jpg*

*Fig. S2.*

***
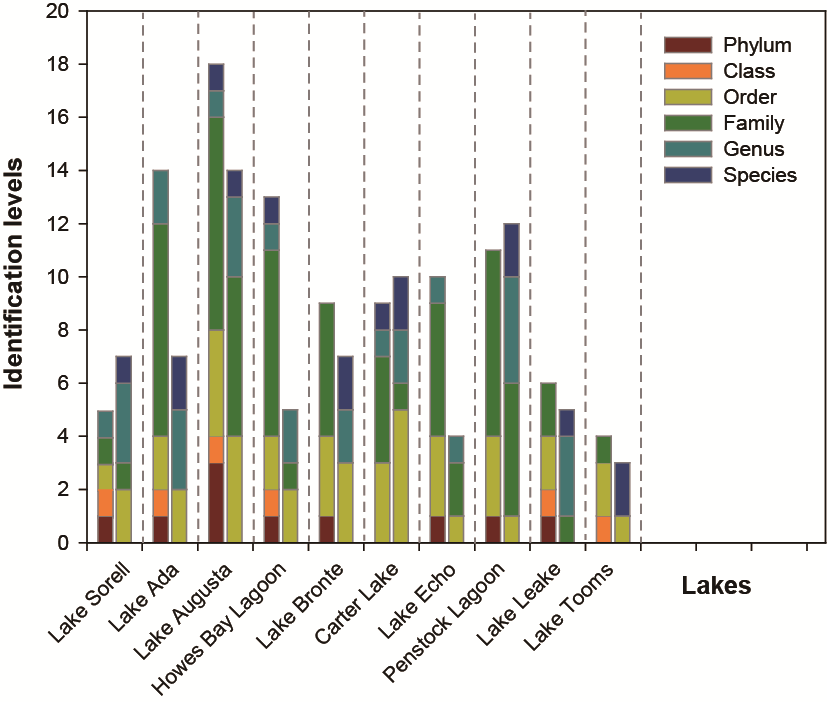
***

*Fig. S3.*


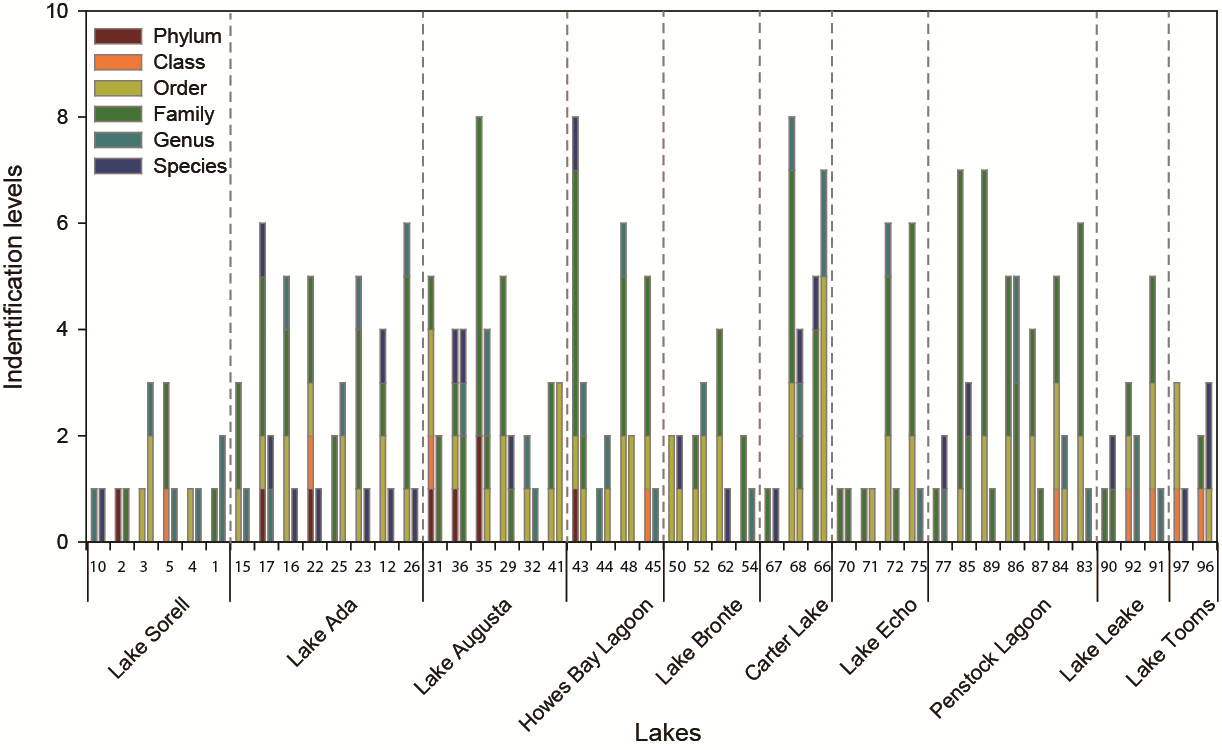


*Fig. S4.*

*Table S1.*

|  | **Storage condition (temperature)** | **Forwarding to** | **Analysis** | **Ethanol removal** | **Date** |
| --- | --- | --- | --- | --- | --- |
| **Tasmania, Australia** | Stored in 96% ethanol  (RT) | Denmark | - | No | 2007 |
| **Silkeborge, Denmark** | Stored in 96% ethanol  (RT) | China | - | Yes  (forwarding) | 2007-2012 |
| **Nanjing, China** | Stored in 96% ethanol  (RT) | S. Korea | Visual inspection | Yes  (analysis and forwarding) | 2012-2013 |
| **Busan,**  **S. Korea** | Freeze-stored in refrigerator  (-80℃) |  | DNA analysis | Yes  (analysis) | 2013-present |
|  |  | 3 times | 2 times | 3 times | 1. years |

*Table S2.*

| **No** | **Sample ID** | | **Length**  **(cm)** | | | **Weight**  **(g)** | **Sampling date** | **Cosmo DNA KIT**  **(1^st^ attempt)** | **Qiazen Stool KIT (2^nd^)** | **Qiazen DNA KIT (3^rd^)** | **PCR success** | **Sequences** |
| --- | --- | --- | --- | --- | --- | --- | --- | --- | --- | --- | --- | --- |
| 1 | T-1-1 | | | 50.1 | | 1425 | 2007-01-13 | 2013-11-12 | 2014-01-19 |  | Success | 1 |
| 2 | T-1-3 | | | 38.1 | | 682 | 2007-01-13 | 2013-11-12 |  |  | Success | 9 |
| 3 | T-1-4 | | | 40.2 | | 697 | 2007-01-13 | 2013-11-23 |  |  | Success | 30 |
| 4 | T-1-6 | | | 48.3 | | 1220 | 2007-01-13 | 2013-11-12 |  |  | Success | 10 |
| 5 | T-1-8 | | | 47.8 | | 1325 | 2007-01-13 | 2013-11-12 |  |  | Success | 15 |
| 6 | T-1-10 | | | 46.8 | | 1005 | 2007-01-13 | 2013-11-12 |  |  | Failure |  |
| 7 | T-1-11 | | | 50.9 | | 1395 | 2007-01-13 | 2013-11-12 |  |  | Failure |  |
| 8 | T-1-23 | | | 27.6 | | 213 | 2007-01-13 | 2013-11-12 |  |  | Failure |  |
| 9 | T-1-24 | | | 49.5 | | 1195 | 2007-01-13 | 2013-11-12 |  |  | Failure |  |
| 10 | T-1-42 | | | 34.8 | | 431 | 2007-01-13 | 2013-11-23 |  |  | Success | 4 |
| 11 | T-1-43 | | | 39.5 | | 662 | 2007-01-13 | 2013-11-12 |  |  | Failure |  |
| **Lake Sorell** | | | | | | **S 42°08.048; E 147°10.148** | | |  | **Subtotal** | | **69** |
| 12 | T-3-64 | | | 30.8 | | 371 | 2007-01-16 | 2013-11-12 |  |  | Success | 10 |
| 13 | T-3-67 | | | 39.2 | | 719 | 2007-01-16 | 2013-11-09 |  |  | Failure |  |
| 14 | T-3-68 | | | 21.9 | | 286 | 2007-01-16 | 2013-11-09 |  |  | Failure |  |
| 15 | T-3-70 | | | 1.6 | | 69 | 2007-01-16 | 2013-11-09 |  |  | Success | 1 |
| 16 | T-3-71 | | | 19.8 | | 85 | 2007-01-16 | 2013-11-09 |  |  | Success | 8 |
| 17 | T-3-74 | | | 16.9 | | 56 | 2007-01-16 | 2013-11-09 |  |  | Success | 13 |
| 18 | T-3-76 | | | 18.6 | | 79 | 2007-01-16 | 2013-11-09 |  |  | Failure |  |
| 19 | T-3-79 | | | 36 | | 537 | 2007-01-16 | 2013-11-23 |  |  | Failure |  |
| 20 | T-3-80 | | | 38 | | 699 | 2007-01-16 | 2013-11-12 |  |  | Failure |  |
| 21 | T-3-82 | | | 19.4 | | 98 | 2007-01-16 | 2013-11-09 |  |  | Failure |  |
| 22 | T-3-83 | | | 21.7 | | 129 | 2007-01-16 | 2013-11-23 |  |  | Success | 6 |
| 23 | T-3-84 | | | 29.4 | | 320 | 2007-01-16 | 2013-11-12 |  |  | Success | 10 |
| 24 | T-3-88 | | | 19.5 | | 90 | 2007-01-16 | 2013-11-09 |  |  | Failure |  |
| 25 | T-3-89 | | | 24.3 | | 178 | 2007-01-16 | 2013-11-23 |  |  | Success | 5 |
| 26 | T-3-90 | | | 38.9 | | 699 | 2007-01-16 | 2013-11-13 |  |  | Success | 8 |
| 27 | T-3-91 | | | 30.2 | | 278 | 2007-01-16 | 2013-11-23 |  |  | Failure |  |
| **Lake Ada** | | | | | | **S 41°52.988; E 146°28.483** | | |  | **Subtotal** | | **61** |
| 28 | T-4-93 | | | 27.2 | | 247 | 2007-01-16 | 2013-11-26 |  |  | Failure |  |
| 29 | T-4-94 | | | 25 | | 185 | 2007-01-16 | 2013-11-13 |  |  | Success | 9 |
| 30 | T-4-95 | | | 19.1 | | 82 | 2007-01-16 | 2013-11-26 |  |  | Failure |  |
| 31 | T-4-96 | | | 14.5 | | 39 | 2007-01-16 | 2013-11-26 |  |  | Success | 10 |
| 32 | T-4-97 | | | 38.7 | | 698 | 2007-01-16 | 2013-11-13 | 2014-01-19 |  | Success | 1 |
| 33 | T-4-99 | | | 25.1 | | 206 | 2007-01-16 | 2013-11-13 |  | 2014-02-28 | Failure |  |
| 34 | T-4-100 | | | 34.8 | | 522 | 2007-01-16 | 2013-11-09 |  | 2014-02-28 | Failure |  |
| 35 | T-4-101 | | | 18 | | 66 | 2007-01-16 | 2013-11-26 |  | 2014-02-28 | Success | 3 |
| 36 | T-4-102 | | | 16.4 | | 49 | 2007-01-16 | 2013-11-26 |  | 2014-02-28 | Success | 15 |
| 37 | T-4-103 | | | 70.4 | | 357 | 2007-01-16 | 2013-11-13 |  | 2014-02-28 | Failure |  |
| 38 | T-4-107 | | | 16.3 | | 56 | 2007-01-16 | 2013-11-26 |  | 2014-02-28 | Failure |  |
| 39 | T-4-108 | | | 16.2 | | 60 | 2007-01-16 | 2013-11-23 |  |  | Failure |  |
| 40 | T-4-109 | | | 18.5 | | 72 | 2007-01-16 | 2013-11-23 |  |  | Failure |  |
| 41 | T-4-110 | | | 44.6 | | 984 | 2007-01-16 | 2013-11-13 |  |  | Success | 10 |
| **Lake Augusta** | | | | | | **S 41°52.208; E 146°30.804** | | |  | **Subtotal** | | **48** |
| 42 | T-5-112 | | | 29.6 | | 379 | 2007-01-18 | 2013-11-26 |  | 2014-02-28 | Failure |  |
| 43 | T-5-114 | | | 38.8 | | 797 | 2007-01-18 | 2013-11-09 |  | 2014-02-28 | Success | 18 |
| 44 | T-5-115 | | | 40.9 | | 913 | 2007-01-18 | 2013-11-23 |  | 2014-02-28 | Success | 9 |
| 45 | T-5-116 | | | 44.6 | | 1125 | 2007-01-18 | 2013-11-13 |  | 2014-02-28 | Success | 10 |
| 46 | T-5-117 | | | 45.7 | | 1230 | 2007-01-18 | 2013-11-07 |  |  | Failure |  |
| 47 | T-5-118 | | | 43.1 | | 946 | 2007-01-18 | 2013-11-23 |  |  | Failure |  |
| 48 | T-5-119 | | | 42.5 | | 977 | 2007-01-18 | 2013-11-23 |  | 2014-02-28 | Success | 15 |
| 49 | T-5-120 | | | 38.7 | | 855 | 2007-01-18 | 2013-11-26 |  |  | Failure |  |
| **HowesBay** | | | | | | **S 41°51.916; E 146°32.813** | | |  | **Subtotal** | | **52** |
| 50 | T-6-131 | | | 17.5 | | 67 | 2007-01-18 | 2013-11-09 |  | 2014-02-28 | Success | 6 |
| 51 | T-6-135 | | | 21.5 | | 122 | 2007-01-18 | 2013-11-09 |  | 2014-02-28 | Failure |  |
| 52 | T-6-137 | | | 27.6 | | 258 | 2007-01-18 | 2013-11-07 |  |  | Success | 5 |
| 53 | T-6-138 | | | 23.9 | | 182 | 2007-01-18 | 2013-11-09 |  | 2014-02-28 | Failure |  |
| 54 | T-6-139 | | | 40.3 | | 661 | 2007-01-18 | 2013-11-23 |  | 2014-02-28 | Success | 1 |
| 55 | T-6-140 | | | 28.1 | | 264 | 2007-01-18 | 2013-11-23 |  | 2014-02-28 | Failure |  |
| 56 | T-6-142 | | | 30.1 | | 368 | 2007-01-18 | 2013-11-23 |  |  | Failure |  |
| 57 | T-6-143 | | | 43.3 | | 773 | 2007-01-18 | 2013-11-23 |  | 2014-02-28 | Failure |  |
| 58 | T-6-145 | | | 32.1 | | 412 | 2007-01-18 | 2013-11-13 |  | 2014-02-28 | Failure |  |
| 59 | T-6-146 | | | 38.4 | | 633 | 2007-01-18 | 2013-11-07 |  |  | Failure |  |
| 60 | T-6-147 | | | 28.3 | | 278 | 2007-01-18 | 2013-11-07 |  |  | Failure |  |
| 61 | T-6-148 | | | 39.2 | | 650 | 2007-01-18 | 2013-11-07 |  |  | Failure |  |
| 62 | T-6-149 | | | 31.8 | | 404 | 2007-01-18 | 2013-11-09 | 2014-01-19 |  | Success | 1 |
| 63 | T-6-150 | | | 30.2 | | 361 | 2007-01-18 | 2013-11-23 |  |  | Failure |  |
| 64 | T-6-153 | | | 19.5 | | 90 | 2007-01-18 | 2013-11-23 |  |  | Failure |  |
| 65 | T-6-154 | | | 33.3 | | 399 | 2007-01-18 | 2013-11-07 |  | 2014-03-06 | Failure |  |
| **Lake Bronte** | | | | | | **S 42°11.200; E 146°28.752** | | |  | **Subtotal** | | **13** |
| 66 | T-7-158 | | | 50.5 | | 1210 | 2007-01-18 | 2013-11-07 |  |  | Success | 18 |
| 67 | T-7-159 | | | 36.5 | | 530 | 2007-01-18 | 2013-11-23 |  |  | Success | 7 |
| 68 | T-7-160 | | | 47.3 | | 961 | 2007-01-18 | 2013-11-23 |  |  | Success | 18 |
| 69 | T-7-164 | | | 49.5 | | 1205 | 2007-01-18 | 2013-11-07 |  |  | Failure |  |
| **Carter Lake** | | | | | | **S 41°51.739; E 146°32.150** | | |  | **Subtotal** | | **43** |
| 70 | T-8-167 | | | 35.3 | | 585 | 2007-01-20 | 2013-11-23 |  |  | Success | 3 |
| 71 | T-8-168 | | | 40.6 | | 766 | 2007-01-20 | 2013-11-23 |  |  | Success | 1 |
| 72 | T-8-173 | | | 36.1 | | 576 | 2007-01-20 | 2013-11-13 |  |  | Success | 1 |
| 73 | T-8-188 | | | 37.6 | | 609 | 2007-01-20 | 2013-11-13 |  |  | Failure |  |
| 74 | T-8-191 | | | 36.9 | | 556 | 2007-01-20 | 2013-11-09 |  | 2014-03-06 | Failure |  |
| 75 | T-8-193 | | | 37.3 | | 654 | 2007-01-20 | 2013-11-09 |  | 2014-03-06 | Success | 1 |
| 76 | T-8-195 | | | 38.2 | | 654 | 2007-01-20 | 2013-11-09 |  | 2014-03-06 | Failure |  |
| **Lake Echo** | | | | | | **S 42°12.935; E 146°38.298** | | |  | **Subtotal** | | **6** |
| 77 | T-9-213 | | | 37.1 | | 684 | 2007-01-21 | 2013-11-23 | 2014-01-19 |  | Success | 2 |
| 78 | T-9-214 | | | 34.5 | | 526 | 2007-01-21 | 2013-11-07 |  | 2014-03-06 | Failure |  |
| 79 | T-9-215 | | | 42.3 | | 941 | 2007-01-21 | 2013-11-23 |  |  | Failure |  |
| 80 | T-9-216 | | | 42 | | 906 | 2007-01-21 | 2013-11-23 |  |  | Failure |  |
| 81 | T-9-217 | | | 50.4 | | 1500 | 2007-01-21 | 2013-11-07 |  | 2014-03-06 | Failure |  |
| 82 | T-9-218 | | | 24.9 | | 222 | 2007-01-21 | 2013-11-23 |  |  | Failure |  |
| 83 | T-9-220 | | | 43.2 | | 1180 | 2007-01-21 | 2013-11-07 | 2014-01-19 |  | Success | 11 |
| 84 | T-9-221 | | | 43.1 | | 1005 | 2007-01-21 | 2013-11-07 |  |  | Success | 10 |
| 85 | T-9-222 | | | 40.1 | | 850 | 2007-01-21 | 2013-11-09 |  | 2014-03-06 | Success | 18 |
| 86 | T-9-223 | | | 40.9 | | 848 | 2007-01-21 | 2013-11-09 |  |  | Success | 20 |
| 87 | T-9-224 | | | 41.2 | | 1045 | 2007-01-21 | 2013-11-09 |  |  | Success | 6 |
| 88 | T-9-225 | | | 52.2 | | 1750 | 2007-01-21 | 2013-11-09 |  |  | Failure |  |
| 89 | T-9-226 | | | 40.4 | | 870 | 2007-01-21 | 2013-11-09 |  | 2014-03-06 | Success | 13 |
| **Penstock lagoon** | | | | | | **S 42°05.614; E 146°45.878** | | |  | **Subtotal** | | **80** |
| 90 | T-13-291 | | | 46.5 | | 1335 | 2007-01-26 | 2013-11-23 |  | 2014-03-06 | Success | 11 |
| 91 | T-13-292 | | | 53.5 | | 1910 | 2007-01-26 | 2013-11-13 |  | 2014-03-06 | Success | 10 |
| 92 | T-13-293 | | | 52.4 | | 1850 | 2007-01-26 | 2013-11-09 | 2014-01-19 |  | Success | 2 |
| 93 | T-13-294 | | | 49.2 | | 1480 | 2007-01-26 | 2013-11-23 |  |  | Failure |  |
| 94 | T-13-308 | | | 49.8 | | 1585 | 2007-01-26 | 2013-11-23 |  |  | Failure |  |
| 95 | T-13-309 | | | 51.2 | | 1670 | 2007-01-26 | 2013-11-09 |  |  | Failure |  |
| **Lake Leake** | | | | | | **S 42° 00.382; E 147°49.443** | | |  | **Subtotal** | | **23** |
| 96 | | T-14-332 | | 41.2 | | 644 | 2007-01-27 | 2013-11-23 |  |  | Success | 11 |
| 97 | | T-14-333 | | 35 | | 462 | 2007-01-27 | 2013-11-13 |  |  | Success | 13 |
| 98 | | T-14-334 | | 32.7 | | 420 | 2007-01-27 | 2013-11-26 |  |  | Failure |  |
| 99 | | T-14-335 | | 21.3 | | 118 | 2007-01-27 | 2013-11-26 |  |  | Failure |  |
| 100 | | T-14-337 | | 45 | | 883 | 2007-01-27 | 2013-11-23 |  |  | Failure |  |
| 101 | | T-14-338 | | 44.2 | | 909 | 2007-01-27 | 2013-11-26 |  |  | Failure |  |
| 102 | | T-14-349 | | 40.7 | | 706 | 2007-01-27 | 2013-11-23 |  |  | Failure |  |
| **Lake Tooms** | | | | | | **S 42°13.426;E 147°47.247** | | |  | **Subtotal** | | **24** |
|  |  | | | | **Success rate (%)** | | | **27.5** | **100.0** | **46.4** | **48.0** | **419** |

*Table S3. 🡪 20150216_Table S3.xlsx*

*Table S4.*

|  | **Average of ND** | **SD** | **N** |
| --- | --- | --- | --- |
| **Lake Sorell** | 0.09 | 0.13 | 6.00 |
| **Lake Ada** | 0.03 | 0.05 | 8.00 |
| **Lake Augusta** | 0.07 | 0.10 | 6.00 |
| **Howes Bay Lagoon** | 0.03 | 0.04 | 3.00 |
| **Lake Bronte** | 0.08 | 0.09 | 4.00 |
| **Carter Lake** | 0.10 | 0.08 | 3.00 |
| **Lake Echo** | 0.00 | 0.00 | 4.00 |
| **Penstock Lagoon** | 0.08 | 0.09 | 7.00 |
| **Lake Leake** | 0.19 | 0.15 | 3.00 |
| **Lake Tooms** | 0.08 | 0.10 | 2.00 |
